# IgG anti-hinge antibodies against IgG4 F(ab’)_2_ fragments generated using pepsin are useful diagnostic markers for rheumatoid arthritis: implications of the possible roles of metalloproteinases and IgG subclasses in generating immunogenic hinge epitopes

**DOI:** 10.1186/s13075-020-02251-7

**Published:** 2020-06-26

**Authors:** Toshiyuki Ota, Shun-ichiro Ota, Ayumi Uchino, Shuji Nagano

**Affiliations:** 1 Center for Rheumatic Diseases, Iizuka Hospital, 3-83 Yoshio-machi, Iizuka-shi, Fukuoka 820-8505 Japan; 2 Department of Laboratory Medicine, Iizuka Hospital, 3-83 Yoshio-machi, Iizuka-shi, Fukuoka 820-8505 Japan; 3 Center for Rheumatic Diseases, Shimonoseki City Hospital, Shimonoseki-shi, Yamaguchi 750-0041 Japan; 4 Department of Internal Medicine (Rheumatic Diseases Division), Iizuka Hospital, 3-83 Yoshio-machi, Iizuka-shi, Fukuoka 820-8505 Japan

**Keywords:** Anti-hinge antibody, Matrix metalloproteinase, IgG4, Rheumatoid factor, ACPA, Rheumatoid arthritis, Extended epitope recognition, Diagnosis

## Abstract

**Background:**

Pepsin agglutinators, discovered over 50 years ago, have been recently referred to as anti-hinge antibodies (AHAs) because of their reaction with the IgG hinge epitope. AHAs have different reactivity for each hinge epitope generated by each protease that cleaves the hinge region at different sites. Moreover, AHAs have different reactivity against different hinge epitopes derived from each IgG subclass even when the same protease is used. Since the expression of matrix metalloproteinase-3 (MMP-3) is enhanced in rheumatoid arthritis (RA), AHA production could also be increased. The purpose of this study was to determine whether the levels of AHAs against IgG hinge epitopes produced by MMP-3 are elevated in RA.

**Methods:**

The serum levels of IgG or IgA AHAs against the IgG1/IgG4 F(ab’)_2_ fragments, generated by either MMP-3 or pepsin, were measured using ELISA in 111 patients with RA and 81 healthy controls (HC). Receiver operating characteristic (ROC) analysis was used for obtaining optimal cutoff values and cutoff values indicating high specificity (> 95%) of the AHA. The targeted epitope of a specific AHA was investigated through inhibition ELISA.

**Results:**

Seven AHAs were statistically higher in RA patients than in HC, except IgG AHA against IgG1 F(ab’)_2_, which was generated by MMP-3 proteolytic cleavage. The areas under the ROC curve were 0.66–0.80, although the sensitivities at high specificity were low (5.4–24.3%). The cumulative number of positive AHAs in each individual was statistically higher in RA patients than in HC, suggesting the extreme extent of AHA repertoires in RA. Inhibition studies revealed that IgG AHAs against IgG4 F(ab’)_2_ fragments generated by pepsin cross-reacted with IgG1 F(ab’)_2_ fragments generated by pepsin. Multivariate logistic regression analysis identified the IgG AHA against IgG4 F(ab’)_2_ fragments generated by pepsin as an independent variable for RA diagnosis, even in RA patients who were negative for both RF and ACPA (odds ratio 1.18, 95% CI 1.06–1.32; *P* = 0.003). Additional experiments using non-RA patients finally strengthened the diagnostic utility.

**Conclusion:**

In RA patients, we observed diversification and amplification of AHA repertoires and diagnostic utility of the specific AHA against IgG4 F(ab’)_2_ fragments generated by pepsin but not MMP-3.

## Background

Rheumatoid arthritis (RA) is a chronic inflammatory disorder characterized by persistent synovitis, destruction of bone and cartilage in multiple joints, and disability [1]. RA is also a systemic autoimmune disease representing autoantibodies, such as rheumatoid factors (RFs; autoantibodies to the IgG Fc fragment), anti-citrullinated protein antibodies (ACPA), and anti-carbamylated protein antibodies [2]. Although RFs are the leading anti-IgG antibodies in RA, antibodies against the IgG hinge epitopes have been also of interest [2].

Multiple proteases cleave IgG at the lower hinge region and produce F(ab’)_2_ fragments [3, 4]. Antibodies to IgG F(ab’)_2_, once designated as pepsin agglutinators, have been detected not only in patients with autoimmune diseases but also in healthy subjects [5, 6]. Lately, they have been termed as anti-hinge antibodies (AHAs), since the majority of the antibodies bind strongly to IgG F(ab’)_2_ and not to intact IgG [7]. Furthermore, AHAs usually target a C terminus of the amino acid sequence in the lower hinge region [8, 9], implying they react specifically to the hinge neoepitope and no other epitopes of IgG F(ab’)_2_.

In recent studies, the reaction of AHAs with human hinge epitopes of human IgG1 or IgG4 appearing after pepsin cleavage showed higher positivity rate and titer in RA patients than in healthy controls (HC) [10]. However, AHAs against IgG1 or IgG4 F(ab’)_2_ generated by other proteases, such as the IgG-degrading enzyme of *Streptococcus pyogens* (IdeS), did not show any difference, suggesting certain AHAs may be associated with a specific disorder.

Matrix metalloproteinase-3 (MMP-3) is produced in synovial cells and has been associated with RA disease activity [11, 12]. It seems likely that excessive production of IgG F(ab’)_2_ fragment in RA is caused by MMP-3, followed by the overproduction of specific AHAs, since activated MMP-3 generates human IgG1 F(ab’)_2_ fragment in vitro and in vivo [3, 13].

The purpose of the present study is to measure IgG/IgA AHAs against IgG1, IgG2, and IgG4 monoclonal therapeutic biologics cleaved by MMP-3 or pepsin and to evaluate their characteristics in RA. To our knowledge, a clinical study of serum AHAs against IgG1 and IgG4 F(ab’)_2_ fragments generated by MMP-3 has never been attempted before.

## Methods

### RA patients, healthy controls (HC), and non-RA patients

In this cross-sectional and case-control study, serum samples were collected from 111 patients with RA who met the ACR classification criteria [14]. As HC, we obtained sera from 81 healthy staffs in our hospital. All sera were leftovers, and there was a shortage of five sera for testing IgA AHAs. All the samples were stored at − 80 °C until use.

Characterizations of the RA patients and the HC are shown in Table 1. The RA patient cohort was significantly older than the HC group, although there were no gender differences between the two groups. Many RA patients had long disease duration and were treated with biologics. Positive for RF and anti-CCP2 antibodies due to routine laboratory examination were 67/111 (60.4%) and 77/111 (69.4%), respectively.

**Table 1:** Characteristics of patients with RA and healthy controls (HC)

	RA (*n* = 111)	HC (*n* = 81)	*P*
Age, years*	63.0 (53.3–66.0)	57.0 (49.8–64.0)	0.004
Women, *n* (%)	91 (82.0)	60 (74.1)	0.21
Duration of disease, years*	5.0 (3.0–12.0)		
bDMARDs^†^, *n* (%)	77 (69.4)		
CDAI*	3.4 (1.4–8.3)		
HAQ score*	0.13 (0–0.75)		
Steinbrocker’s stage, *n* (%)			
I	18 (16.2)		
II	35 (31.5)		
III	15 (13.5)		
IV	43 (38.7)		
Smoking, *n* (%)	34 (30.6)	NA	
Autoantibodies, *n* (%)			
RF (+)/RF (−)	67 (60.4)/44 (39.6)	NA	
Anti-CCP2 (+)/anti-CCP2 (−)/NA	77 (69.4)/28 (25.2)/6 (5.4)	NA	

*NA* not available. *Values are the median (interquartile range) unless otherwise indicated. ^†^Among bDMARDs, tocilizumab was administered in 30 patients

Furthermore, sera from 61 patients (50 female, 17–83 years old; 11 male, 48–84 years old) with non-RA rheumatic diseases were used for measurement of AHAs. The non-RA was composed of SLE (*n* = 22), polymyositis/dermatomyositis (*n* = 9), Behcet disease (*n* = 5), polymyalgia rheumatica (*n* = 4), primary Sjögren’s syndrome (*n* = 3), limited cutaneous systemic sclerosis (*n* = 3), ANCA-associated vasculitis (*n* = 3), MCTD (*n* = 2), adult-onset Still’s disease (*n* = 2), Takayasu’s arteritis (*n* = 1), IgG4-related disease (*n* = 1), polyarteritis nodosa (*n* = 1), psoriatic arthritis (*n* = 1), RS3PE (*n* = 1), SAPHO syndrome (*n* = 1), sarcoidosis (*n* = 1), and acute transient arthritis (*n* = 1).

### Generation of F(ab’)_2_ fragments by proteolytic cleavage

The following biologics were used: tocilizumab (TCZ;IgG1), infliximab (IFX;IgG1), panitumumab (PAN;IgG2), and natalizumab (NTZ;IgG4). All biologics, except IFX, were humanized monoclonal IgG. Although IFX is chimeric, the hinge region and CH2 domain were derived from human IgG1 according to the database from the National Center for Biotechnology Information (NCBI, USA). We used pepsin (Sigma-Aldrich, St. Louis, USA) and human MMP-3 as proteases to cleave the biologics. The pro-MMP-3 was a kind gift from Daiichi-Fine Chemical, Toyama, Japan.

The biologics were dialyzed with 0.1 M sodium citrate buffer (SCB, pH 3.5), and then 100 μg of pepsin in SCB was incubated at 37 °C with 10 mg of each biologic. Incubation time was overnight except 2 h for PAN. Tris 1 M was added to the IgG solution until the pH increased to 7.4 to stop the digestion. Proteolysis by MMP-3 was performed after the activation of pro-MMP-3 by incubation at 55 °C for 25 min [15]. The activated MMP-3 (50 μg) in 50 mM Tris-HCl (pH 7.5) containing 150 mM NaCl and 10 mM CaCl_2_ was mixed with 5 mg of each biologic at 37 °C. After 2 h of incubation, each small amount of the reaction mixture (20 μL) was removed, and the reaction was stopped by rapid freezing. The remaining reaction mixture was continued for 24 h and stopped by adjustment to 20 mM ethylenediaminetetraacetic acid (EDTA).

### Detection of human IgG fragments

Cleaved human IgG fragments were analyzed by SDS-PAGE in Tris-glycine buffer using 10% gels under non-reducing conditions. Samples were heated at 100 °C for 2 min in 50 mM Tris-HCl buffer (pH 6.8) with a final concentration of 20% glycerin and 1% (w/v) SDS. Protein bands were visualized by staining with 0.25% (w/v) Coomassie Brilliant Blue R-250 (Nacalai Tesque, Kyoto, Japan) in 50% (v/v) methanol and 10% (v/v) acetic acid.

### Purification of IgG F(ab’)_2_ fragments

At the outset, human IgG digested by pepsin or MMP-3 was separated by gel filtration on a Sephadex G-150 column (Pharmacia Fine Chemicals, Uppsala, Sweden). The estimated IgG F(ab’)_2_ fractions were concentrated by Vivaspin 20 (Sartorius Stedium, Goettingen, Germany). Finally, to remove IgG possessing Fc, the concentrated crude IgG F(ab’)_2_ were applied to a Protein G Mag Sepharose column (GE Healthcare, Uppsala, Sweden) using 50 mM Tris buffer with 150 mM NaCl at pH 7.5. Purification of the IgG F(ab’)_2_ was confirmed by the SDS-PAGE described above. Each purified F(ab’)_2_ fragment was denoted by the addition of an italic subscript to specify the protease responsible for its cleavage, e.g., IgG1 F(ab’)_2*MMP-3*_.

### Measurement of AHAs by enzyme-linked immunosorbent assay (ELISA)

ELISA plates (Sumitomo Bakelite, Tokyo, Japan) were coated overnight at 4 °C with 100 μL/well of a solution of 0.5 μg/ml IgG F(ab’)_2_ in 0.1 M carbonate/bicarbonate buffer at pH 9.6. After washing with 10 mM Tris buffer containing 0.9% NaCl with 0.05% Tween-20 (TBST) at pH 7.4, serum samples diluted 1:200 with TBST were added to the plate (100 μL/well). These were incubated for 2 h at room temperature (RT). After washing, 100 μL/well of alkaline phosphatase (ALP)-conjugated anti-human IgG Fc (Sigma-Aldrich) diluted 1:10,000 with TBST, or ALP-conjugated anti-human IgA (Sigma-Aldrich) diluted 1:10,000 with TBST was added and incubated for 1 h at RT. After washing, the AHAs were visualized with 1 mg/mL of p-nitrophenyl phosphate tablets (Sigma-Aldrich) in diethanolamine buffer at pH 9.8 for 30 min or for 2 h for IgA AHA measurement. Absorbance was measured at 405 nm using a microplate reader.

Levels of IgG AHA were calculated by a calibration curve using pooled human IgG purified using 40% ammonium sulfate and DEAE sephadex (Pharmacia Fine Chemicals, Uppsala, Sweden). We arbitrarily defined 1 mg/mL of the pooled IgG as containing 800 arbitrary units (AU)/mL of IgG AHA against IgG F(ab’)_2*pepsin*_. We also used this calibration curve for measuring IgG or IgA AHA to other IgG F(ab’)_2_ fragments.

### Inhibition study for specificities of IgG AHAs against IgG1/IgG4 F(ab’)_2*pepsin*_

An equal volume of inhibitors with various concentrations (5, 000, 1, 000, 200, 40, 8, 1.6, 0.32, 0 μg/mL) and 1:200 diluted IgG AHA-positive serum from RA patients were thoroughly mixed, followed by incubation for 2 h at RT. The mixtures were added to the ELISA plate (100 μL/well) coated with IgG1/IgG4 F(ab’)_2*pepsin*_ and then allowed to react for 2 h at RT. The subsequent procedure was the same as the measurement of AHA described above. The extents of inhibition were expressed as percent inhibition of the AHA responses, calculated as follows:

$$ \%\mathrm{inhibition}=\left(\frac{\mathrm{B}-\mathrm{A}\ }{\mathrm{B}}\right)\times 100 $$

*A* is the absorbance in the presence of inhibitors and *B* absorbance in the absence of inhibitors.

### Statistical analysis

We used the Mann-Whitney *U* test and the Kruskal-Wallis test to compare the differences between two groups and among multiple groups, respectively. We also used Fisher’s exact and *χ*^2^ test for nominal characteristic. To elucidate the independent variables associated with RA diagnosis, univariate logistic regression followed by multivariate logistic regression analysis was performed. Age and gender added to the model. A two-tailed *P* < 0.05 was considered statistically significant. Data were analyzed on a personal computer using SPSS version 19 (IBM Japan, Tokyo, Japan) and Statflex version 6 (Artech, Osaka, Japan).

## Results

### Cleavage of the biologics by MMP-3

Monoclonal IgG1 (TCZ, IFX), IgG2 (PAN), and IgG4 (NTZ) were cleaved by MMP-3, and the products were analyzed using non-reducing SDS-PAGE (Additional file 1: Figure S1). To visualize the generated human IgG1 fragments, we have provided their schematic diagrams (shown in Additional file 1: Figure S2). We obtained the purified IgG1 F(ab’)_2*MMP-3*_ and IgG4 F(ab’)_2*MMP-3*_.

### Different levels of serum AHAs against various IgG F(ab’)_2_

**Fig. 1:**
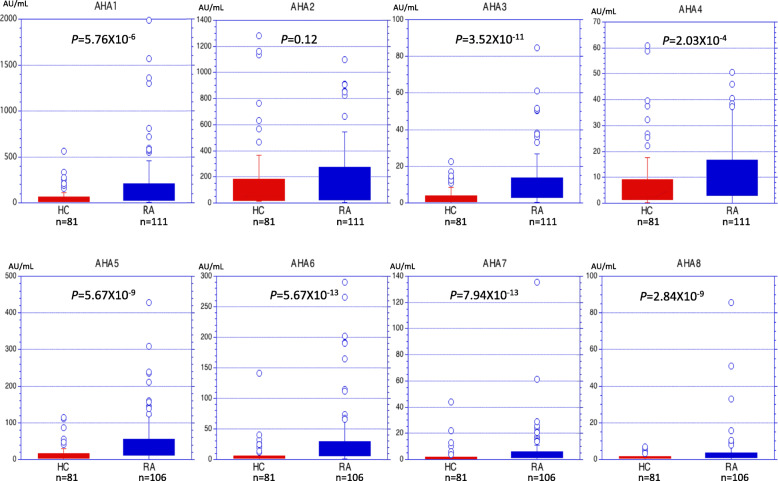
Comparison of AHA levels between HC and RA patients. AHA1, AHA2, AHA3, and AHA4 represent IgG AHA against TCZ IgG1 F(ab’)_2*pepsin*_, TCZ IgG1 F(ab’)_2*MMP-3*_, NTZ IgG4 F(ab’)_2*pepsin*_, and NTZ IgG4 F(ab’)_2*MMP-3*_, respectively. AHA5, AHA6, AHA7, and AHA8 represent IgA AHAs against TCZ IgG1 F(ab’)_2*pepsin*_, TCZ IgG1 F(ab’)_2*MMP-3*_, NTZ IgG4 F(ab’)_2*pepsin*_, and NTZ IgG4 F(ab’)_2*MMP-3*_, respectively. Differences in levels between HC and RA were analyzed with the Mann-Whitney *U* test

Levels of serum AHAs against the four different IgG F(ab’)_2_, excluding AHA2 (IgG anti-TCZ IgG1F(ab’)_2*MMP-3*_), were significantly higher in RA patients (*n* = 111 or 106) than in HC (*n* = 81; Fig. 1). Median values of IgG anti-IgG1 F(ab’)_2_ (AHA1, AHA2) were about 8–10 times higher than those of IgG anti-IgG4 F(ab’)_2_ (AHA3, AHA4). Furthermore, when the same F(ab’)_2_ antigen was used, median values of IgG AHAs were 2–6 times higher than those of IgA AHAs (e.g., AHA1 vs. AHA5). Among the IgG AHAs, AHA3 had the highest discriminative power (*P* = 3.52 × 10^−11^). In general, all IgA AHAs (AHA5–AHA8) seemed to be superior to IgG AHAs as to discriminating between RA and HC.

**Fig. 2:**
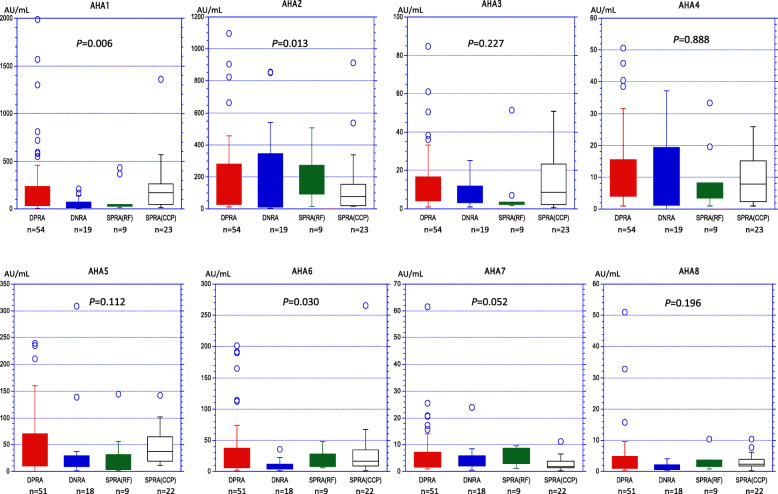
Comparison of AHA levels in RA stratified by the presence or absence of RF and anti-CCP2 antibodies (CCP). Double positive RA (DPRA) indicates RA patients being positive for both RF and CCP. Double negative RA (DNRA) indicates RA patients being neither. Single positive RA (SPRA) indicates RA patients being either positive for RF [SPRA (RF)] or positive for CCP [SPRA (CCP)]. The number of patients in each RA group is shown. The non-parametric Kruskal-Wallis test was used to compare the 4 groups

AHA levels in RA patients were compared after stratification according to positive/negative for RF and anti-CCP2, namely double positive RA (DPRA), double negative RA (DNRA), RA with single positive RF (SPRA (RF)), and RA with single positive anti-CCP2 (SPRA (CCP)). As shown in Fig. 2, the Kruskal-Wallis test revealed significant AHA level differences among the four groups in AHA1, AHA2, and AHA6. Moreover, the Mann-Whitney *U* test was used to compare the differences between the groups, and the AHA levels of the DNRA in AHA, AHA2, and AHA6 were significantly lower than those of the DPRA (AHA1, *P* = 0.004; AHA2, *P* = 0.003; AHA6, *P* = 0.03).

### Specificity of IgG AHA responses against the F(ab’)_2*pepsin*_ of IgG1 or IgG4

**Fig. 3:**
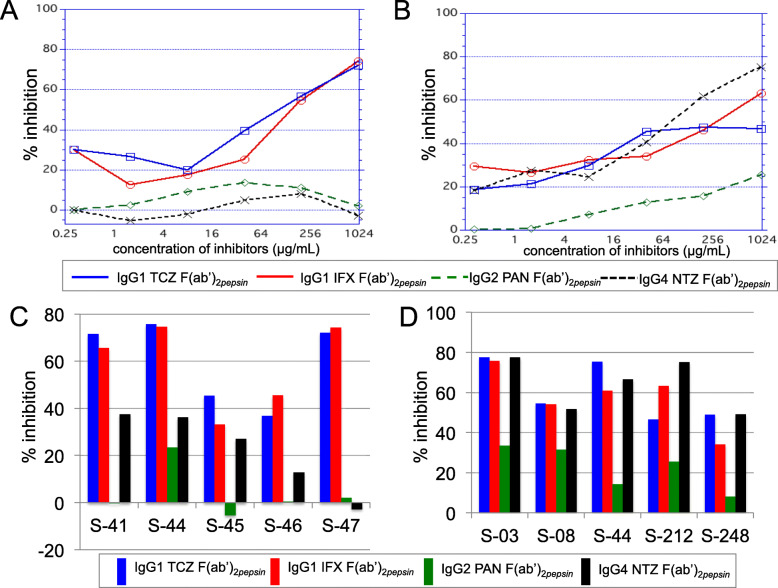
Inhibition studies for hinge epitope specificity of IgG AHA. Typical cases were shown in **a** and **b**. **a** IgG AHA from RA patient (S-47) against IgG1 TCZ F(ab’)_2*pepsin*_ was neither inhibited by IgG2 PAN nor IgG4 NTZ F(ab’)_2*pepsin*_, but in a dose-dependent manner by both TCZ-and IFX F(ab’)_2*pepsin*_. **b** IgG AHA from RA patient (S-212) against IgG4 F(ab’)_2*pepsin*_ was inhibited in a dose-dependent manner by IgG4 F(ab’)_2*pepsin*_, followed by IgG1 IFX or TCZ F(ab’)_2*pepsin*_, and slightly by IgG2 F(ab’)_2*pepsin*_. **c** Percent inhibitions of IgG AHAs from five RA patients (S-41, S-44, S-45, S-46, S-47) against IgG1 TCZ F(ab’)_2*pepsin*_ by inhibitors (four different IgG F(ab’)_2*pepsin*_) at 1000 μg/mL were 36.8–75.6 for TCZ F(ab’)_2*pepsin*_, 33.2–74.5 for IFX F(ab’)_2*pepsin*_, − 5.4–23.4 for PAN F(ab’)_2*pepsin*_, and − 2.8–37.4 for NTZ F(ab’)_2*pepsin*_. **d** When IgG4 NTZ F(ab’)_2*pepsin*_ was used as coating antigen and four inhibitors at 1000 μg/mL were used, percent inhibitions of IgG AHAs from RA patients (S-03, S-08, S-44, S-212, S-248) were 46.6–77.5 for TCZ, 34.2–75.8 for IFX, 8.2–33.5 for PAN, and 49.2–77.6 for NTZ

To elucidate whether IgG AHA responses against the F(ab’)_2*pepsin*_ of IgG1 or IgG4 possess a specificity for epitopes on each IgG subclass F(ab’)_2*pepsin*_, inhibition studies were implemented. The response of serum AHAs against IgG1 TCZ F(ab’)_2*pepsin*_ from a patient with RA (S-47) was not inhibited by neither IgG2 nor IgG4 F(ab’)_2*pepsin*_, but it was inhibited in a dose-dependent manner by IgG1 F(ab’)_2*pepsin*_ (Fig. 3a). Meanwhile, the AHA response against IgG4 F(ab’)_2*pepsin*_ was inhibited by not only IgG4 F(ab’)_2*pepsin*_, but also IgG1 F(ab’)_2*pepsin*_ to a certain extent (Fig. 3b) in another RA patient (S-212). These results indicate the possibility that IgG AHAs against IgG1 F(ab’)_2*pepsin*_ specifically react with IgG1 F(ab’)_2*pepsin*_, but those against IgG4 F(ab’)_2*pepsin*_ cross-react with IgG1 F(ab’)_2*pepsin*_. To further ascertain the possible properties, percent inhibitions at a defined inhibitor concentration (1000 μg/mL) were calculated in sera from nine RA patients. IgG AHAs against IgG1 F(ab’)_2*pepsin*_ exhibited a predisposition to react to pepsin-digested IgG1 hinge neoepitopes (Fig. 3c). IgG1 F(ab’)_2*pepsin*_, however, inhibited the reactions of five IgG AHAs against IgG4 F(ab’)_2*pepsin*_ to the same extent as IgG4 F(ab’)_2*pepsin*_ (Fig. 3d). These observations indicated that AHAs against pepsin-digested IgG4 had a tendency to cross-react with IgG1 F(ab’)_2*pepsin*_; meanwhile, specificity of AHAs against IgG1 F(ab’)_2 *pepsin*_ was mostly restricted to IgG1 F(ab’)_2*pepsin*_.

### Use of AHAs for clinical RA testing

We assessed the clinical usefulness of AHAs for RA diagnosis by ROC curve analysis. The values of the area under the curve and their mean standard error (SE) (Table 2) ranged from 0.57 (0.04; AHA2) to 0.80 (0.03; AHA6 and AHA7). After the optimal cutoff values of each AHA were calculated by Youden’s index, we obtained sensitivity/specificity, positive predictive value (PPV+)/negative predictive value (PPV−), positive likelihood ratio (LR+), and diagnostic odds ratio (DOR) as shown in condition 1 of Table 2. The LR+ values were 1.79 (95% CI 1.23–2.62) of AHA1 to 3.44 (95% CI 2.69–4.11) of AHA3, leading to only a small shift in pre- and post-test probability. The DOR of AHA6 and AHA7 were relatively high, yielding 10.24 (95% CI 5.40–19.42) and 11.35 (95% CI 5.65–22.79), respectively. Next, the same statistical indices were calculated at high specificity conditions (96–98%) for RA as shown in condition 2 of Table 2. Increments of the LR+ values, except for those in AHA2 and AHA4, resulted in moderate shifts in pre- and post-test probability, although the values of sensitivity were as low as 2.7% for AHA4 and 24.3% for AHA3. The DOR values also changed (e.g., from 7.02 to 12.7 of AHA3 and 11.4 to 4.57 of AHA7).

**Table 2:** Evaluation of AHA at optimal cutoff value (condition 1) and at cutoff value for high specificity (condition 2)

	Cutoff	AUC (SE)	Sen/Spe	PPV+/PPV−	LR+ (95% CI)	DOR (95% CI)
Condition 1						
AHA1	22.5	0.69 (0.04)	82.0/54.3	71.1/68.8	1.79 (1.23–2.62)	5.41 (2.89–10.1)
AHA2	204	0.57 (0.04)	29.7/86.4	75.0/47.3	2.19 (1.74–2.75)	2.69 (1.29–5.63)
AHA3	4.70	0.78 (0.03)	59.5/82.7	82.5/59.8	3.44 (2.69–4.41)	7.02 (3.66–14.5)
AHA4	4.10	0.66 (0.04)	70.3/65.4	73.6/61.6	2.03 (1.52–2.72)	4.47 (2.46–8.13)
AHA5	10.3	0.74 (0.04)	78.3/66.7	75.5/70.1	2.35 (1.64–3.36)	7.22 (3.86–13.5)
AHA6	5.40	0.80 (0.03)	79.3/72.8	79.3/72.8	2.92 (2.02–4.22)	10.2 (5.40–19.4)
AHA7	1.20	0.80 (0.03)	89.6/56.8	73.1/80.7	2.07 (1.21–3.56)	11.4 (5.65–22.8)
AHA8	1.10	0.74 (0.04)	73.6/67.9	75.0/66.3	2.29 (1.66–3.16)	5.89 (3.19–10.9)
Condition 2						
AHA1	277	0.69 (0.04)	16.2/97.5	90.0/45.9	6.57 (5.37–8.03)	7.65 (2.10–27.9)
AHA2	766	0.57 (0.04)	5.40/96.3	66.7/42.6	1.46 (0.90–2.36)	1.49 (0.36–6.08)
AHA3	14.8	0.78 (0.03)	24.3/97.5	93.1/48.5	9.85 (8.24–11.8)	12.7 (3.85–41.8)
AHA4	39.7	0.66 (0.04)	2.70/97.5	60.0/42.3	1.10 (0.53–2.26)	1.10 (0.18–6.72)
AHA5	89.8	0.74 (0.04)	16.0/97.5	89.5/47.0	6.50 (5.27–8.01)	7.55 (2.05–27.7)
AHA6	32.5	0.80 (0.03)	23.6/97.5	92.6/49.4	9.55 (7.93–11.5)	12.2 (3.66–40.6)
AHA7	12.6	0.80 (0.03)	10.4/97.5	84.6/45.4	4.20 (3.21–5.50)	4.57 (1.11–18.8)
AHA8	5.37	0.74 (0.04)	16.0/97.5	89.5/47.0	6.50 (5.27–8.01)	7.55 (2.05–27.7)

The cutoff values represent as AU/mL. *AUC* area under the curve, *SE* standard error, *Sen/Spe* sensitivity/specificity, *PPV+/PPV*− positive predictive value/negative predictive value, *LR+* positive likelihood ratio, *95% CI* 95% confidence interval, *DOR* diagnostic odds ratio, *AHA1* IgG anti-TCZ IgG1 F(ab’)_2*pepsin*_, *AHA2* IgG anti-TCZ IgG1 F(ab’)_2*MMP-3*_, *AHA3* IgG anti-NTZ IgG4 F(ab’)_2*pepsin*_, *AHA4* IgG anti-NTZ IgG4 F(ab’)_2*MMP-3*_, *AHA5* IgA anti-TCZ IgG1 F(ab’)_2*pepsin*_, *AHA6* IgA anti-TCZ IgG1 F(ab’)_2*MMP-3*_, *AHA7* IgA anti-NTZ IgG4 F(ab’)_2*pepsin*_, *AHA8* IgA anti-NTZ IgG4 F(ab’)_2*MMP-3*_

### Expansion and diversification of the AHA response in RA

**Fig. 4:**
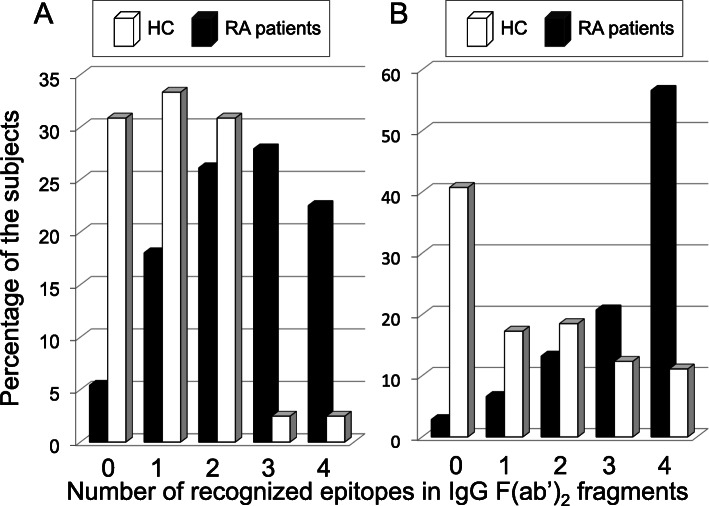
Frequency distribution of cumulative number of hinge epitopes recognized by IgG AHA (**a**) or IgA AHA (**b**) from each patient with RA or each HC. Depicted are the percentages of RA patients and HC

To assess the expansion of the AHA response in RA, we studied how many positive AHAs were obtained within each RA patient or HC (Fig. 4). The AHA exceeding each optimal cutoff value was accepted as positive, and the cumulative number of positive AHA response in RA patients and HC was counted. The IgG and IgA AHAs were classified into five grades (0–4) according to the cumulative number. The grade of IgG AHAs in RA showed a tendency to be higher than that in HC (Fig. 4a). Furthermore, the highest grade (cumulative number of recognized epitopes, 4) of IgA AHAs was over 50% in RA, indicating an extensive recognition profile (Fig. 4b). These results suggested that the AHA repertoire in RA is expanded and diversified.

### Potential of AHAs as independent diagnostic markers for RA

We performed a logistic regression analysis to further assess the potential ability of AHAs as diagnostic markers for RA. Univariate analysis using 106 RA patients and 81 HC showed that AHAs, except AHA2, could be selected for multivariate analysis as shown in analysis 1 of Table 3. A multivariate logistic regression analysis revealed that AHA3 (IgG AHA against NTZ IgG4 F(ab’)_2*pepsin*_) and AHA5 (IgA AHA against TCZ IgG1 F(ab’)_2*pepsin*_) were selected as independent variables contributing to RA diagnosis. Additionally, logistic regression analysis using AHAs of 18 patients with DNRA revealed that only AHA3 was selected as an independent variable (odds ratio 1.18, 95% CI 1.06–1.32; *P* = 0.003) as shown in analysis 2 of Table 3.

**Table 3:** Univariate and multivariate logistic regression analyses of the association between AHA and RA

Variable	Univariate analysis		Multivariate analysis	
	OR (95% CI)	*P*	OR (95% CI)	*P*
Analysis 1: total RA vs. HC				
Age	1.03 (0.99–1.06)	0.075	1.04 (1.00–1.09)	0.037
Gender	1.59 (0.79–3.17)	0.189	2.19 (0.86–5.62)	0.100
AHA1	1.01 (1.00–1.01)	0.001	1.00 (0.99–1.00)	0.992
AHA2	1.00 (0.99–1.00)	0.295	–	–
AHA3	1.18 (1.09–1.27)	1 × 10^−5^	1.16 (1.07–1.26)	3 × 10^−4^
AHA4	1.03 (1.00–1.06)	0.025	1.00 (0.96–1.04)	0.825
AHA5	1.03 (1.02–1.05)	8 × 10^−5^	1.02 (1.00–1.03)	0.034
AHA6	1.06 (1.03–1.09)	4 × 10^−4^	1.01 (0.98–1.04)	0.367
AHA7	1.16 (1.05–1.28)	0.004	1.04 (0.96–1.13)	0.339
AHA8	1.65 (1.30–2.09)	3 × 10^−4^	1.20 (0.89–1.62)	0.226
Analysis 2: DNRA vs. HC				
Age	1.04 (0.98–1.11)	0.226	1.07 (0.98–1.15)	0.115
Gender	2.62 (0.59–13.21)	0.193	9.29 (1.01–85.3)	0.049
AHA1	0.99 (0.99–1.01)	0.898	–	–
AHA2	1.00 (0.99–1.00)	0.243	–	–
AHA3	1.16 (1.06–1.27)	0.002	1.18 (1.06–1.32)	0.003
AHA4	1.03 (0.99–1.07)	0.112	1.03 (0.98–1.08)	0.311
AHA5	1.01 (0.99–1.03)	0.062	1.01 (0.99–1.03)	0.186
AHA6	1.00 (0.98–1.03)	0.664	–	–
AHA7	1.06 (0.98–1.15)	0.143	–	–
AHA8	1.21 (0.83–1.70)	0.347	–	–

The number of RA patients in analysis 1 and in analysis 2 was 106 and 18, respectively. The number of HC was same as 81 in both analyses

To strengthen the potential of AHAs as independent markers for RA, we performed additional experiments using sera from 61 patients with rheumatic diseases excluding RA (non-RA) and sera from 59 patients with RA. The Mann-Whitney *U* test displayed that AHA3 level in RA was significantly higher than that in non-RA (*P* = 2.69 × 10^−4^, Additional file 1: Figure S3). Finally, logistic regression analyses revealed that only AHA3 was selected as an independent marker for RA diagnosis (Table 4).

**Table 4:** Univariate and multivariate logistic regression analyses of the association between AHA and RA when patients with non-RA were used as a subject

Variable	Univariate analysis		Multivariate analysis	
	OR (95% CI)	*P*	OR (95% CI)	*P*
Age	1.01 (0.98–1.03)	0.62	1.00 (0.97–1.03)	0.89
Gender	0.78 (0.32–1.91)	0.58	0.57 (0.21–1.54)	0.27
AHA1	1.00 (0.99–1.01)	0.22	1.00 (0.99–1.00)	0.90
AHA3	1.08 (1.03–1.12)	3.3 × 10^−4^	1.08 (1.03–1.13)	9.9 × 10^−4^
AHA5	1.01 (0.99–1.01)	0.10	1.00 (0.99–1.02)	0.41
AHA7	1.05 (0.98–1.12)	0.15	0.98 (0.90–1.08)	0.73

The numbers of patients with RA and non-RA were 59 and 61, respectively. All of non-RA patients including six patients with Sjogren’s syndrome (3 primary and 6 secondary) did not show persistent destructive polyarthritis, namely RA-like symptom or sign

## Discussion

There have been conflicting reports concerning the specificity of anti-F(ab’)_2_ antibodies in human sera [5, 16, 17]. Nowadays, studies using human monoclonal IgG1 F(ab’)_2_ and synthetic peptide analogs of IgG1 hinge region has revealed that most anti-IgG F(ab’)_2_ antibodies target lower hinge epitopes but not idiotopes nor other epitopes in the IgG F(ab’)_2_ fragment [9, 10, 18]. However, as 30 patients with RA in this study were receiving or had been receiving TCZ, it is not straightforward to clarify whether anti-TCZ IgG F(ab’)_2_ antibodies target idiotopes or other epitopes except lower hinge epitopes. We once tried to detect the anti-idiotype antibodies by ELISA using TCZ IgG F(ab’)_2_ fragment as coating antigen in RA patients who were receiving TCZ. Whereas many anti-TCZ IgG F(ab’)_2_ antibodies were detected, the antibodies were not inhibited by intact TCZ, but only by TCZ IgG F(ab’)_2_ fragment. From these experimental findings, it is likely that most of anti-IgG F(ab’)_2_ antibodies in this study do not react with idiotopes in the biologics.

Serum AHAs exist in healthy individuals and patients with a variety of diseases [19–23]. Notably, results of higher positive incidence and levels of serum AHAs in RA compared with HC have been reported [5, 6, 24], which are in accordance with our results. Contrary to our expectations, IgG AHAs against IgG1 F(ab’)_2*MMP-3*_ were not significantly higher in RA patients than HC, although IgA AHAs against IgG1 F(ab’)_2*MMP-3*_ and, moreover, both IgG and IgA AHA against IgG4 F(ab’)_2*MMP-3*_ were significantly elevated in RA patients. Given the significantly elevated levels of IgA AHAs against IgG1 F(ab’)_2*MMP-3*_ and IgG/IgA AHA against IgG4 F(ab’)_2*MMP-3*_ in RA, it seems likely that upregulated immune responses by IgG1/IgG4 F(ab’)_2*MMP-3*_ fragments are characteristic of RA. However, many questions remain, including why the levels of IgG AHA against IgG1 F(ab’)_2*MMP-3*_ did not show any significant difference, whether there exists an upregulated mechanism associated with increased IgG AHAs in HC, whether the MMP-12 that could cleave IgG1 at the same location in the lower hinge as does MMP-3 [7] could participate in increased AHA production in HC, and if the proteases involved in the generation of IgG1 F(ab’)_2*MMP-3*_ fragments upregulated the production of IgG AHAs continuously. As of now, however, we cannot clearly answer these questions. Remarkably, all IgA AHAs were significantly higher in RA patients than in HC, which might reflect abnormal conditions of mucosal immunity resulting from dysbiosis of the respiratory, gut, and oral mucosa in RA [25, 26].

In regard to the epitopes targeted by AHAs in HC, Falkenburg et al. reported that IgG AHAs against IgG1 F(ab’)_2*pepsin*_ were inhibited by IgG1 F(ab’)_2*pepsin*_ but not by IgG4 F(ab’)_2*pepsin*_, despite the inhibition of IgG AHAs against IgG4 F(ab’)_2*pepsin*_ by both F(ab’)_2*pepsin*_ fragments [10]. These findings partially agree with our inhibition study results using RA patients as shown in Fig. 3. The authors also reported that the specificity of AHA against IgG4 F(ab’)_2*pepsin*_ was clearly different between HC and RA patients, namely, that the AHAs in HC cross-reacted with IgG1 F(ab’)_2*pepsin*_, whereas the AHA in RA patients were only inhibited by IgG4 F(ab’)_2*pepsin*_. Unlike their findings, AHA responses against IgG4 F(ab’)_2*pepsin*_ showing cross-reactivity with IgG1 F(ab’)_2*pepsin*_ were recognized in RA. We suspect this discrepancy originates from using different methods to detect AHAs, namely, our direct coating of IgG F(ab’)_2_ to the ELISA plate compared with their indirect coating, which results in stable and conformational hinge epitope, i.e., anti-biotin IgG F(ab’)_2_ bound to biotinylated human serum albumin.

We found a more extended hinge epitope recognition profile in RA compared with HC. This finding might indicate the possibility of an epitope-spreading phenomenon in which the immune response extends to involve new intramolecular or intermolecular epitopes [27–29], although our study was cross-sectional. This phenomenon has been revealed by the observation of ACPA in RA, which occurs before clinical disease onset [30, 31]. Further studies using preclinical and longitudinal RA patients are needed to confirm whether the same phenomenon is observed.

Compared with ACPA, AHAs were not useful for RA diagnosis because their LR+ values were less than 5 at optimal cutoff values, with anti-CCP2 LR+ values of 71.6 and 12.1 in HC and non-RA patients, respectively [32]. Meanwhile, the LR+ of RFs was not high, reported to be 4.86 [33], and seemed to approximate to the LR+ of AHAs. At a cutoff value of over 95% specificity, however, two AHAs (AHA3 and AHA6) revealed LR+ close to 10, which can lead to an increased probability of RA diagnosis.

Three AHAs (AHA3, AHA5, AHA8) were selected by univariate analysis, and then AHA3 (IgG anti-IgG4 F(ab’)_2*pepsin*_) and AHA 5 (IgA anti-IgG1 F(ab’)_2*pepsin*_) were selected as independent variables for RA diagnosis by multivariate logistic regression analysis as shown in Table 3. Additionally, only AHA3 was selected as an independent variable to conduct the multivariate analysis on DNRA. Furthermore, when patients with non-RA were subject as a substitute for HC, it strengthened a possibility of the conclusion that AHA3 was selected again as shown in Table 4. The resulting increased AHA reactivity against IgG4 F(ab’)_2*pepsin*_ in RA highlights that (1) IgG4 is produced in the context of prolonged antigenic stimulation and (2) AHAs must be generated in vivo by other physiological proteases, except pepsin, since pepsin needs activation in acidic stomach conditions and do not reach circulation. In this regard, activated matrix metalloproteinase-7 (MMP-7) seems to be a candidate protease for human IgG cleavage so that it cleaves IgG4 at the same lower hinge site between F234 and L235 (EU numbering) as pepsin does [10, 13]. Interestingly, the increase in MMP-7 seems to mainly originate from non-articular but extra-articular lesions, such as the nodules and lung of RA patients [12, 34, 35].

Although we cannot clearly explain the role of AHAs in RA, it has been proposed that several biological functions of AHAs, such as B cell suppression due to cross-linking the B cell receptor and FcγRIIb, complement amplification via the capture of dimeric C3b due to immune-complex formation of antigen-binding IgG F(ab’)_2_ and AHA, and the functional restoration of cleaved IgGs without Fc [7]. A possible suggestion in RA is IgG4 ACPA, the leading IgG subclass following IgG1 [36]. IgG4 ACPA would enervate the IgG1-mediated ACPA-associated pathogenic progression through activating the complement system and triggering Fcγ receptors. In this situation, it has been proposed that specific AHAs bind to IgG4 F(ab’)_2_ with ACPA reactivity, form immune complexes, and potentially lead to the progression of inflammatory processes [10]. Further studies are needed to evaluate the pathogenic or protective participation of AHA in addition to RF and ACPA in joints and/or lungs of RA patients.

## Conclusions

We have discovered an extended epitope recognition profile of AHAs in RA, suggesting maturation of AHA-producing immune cells. IgG AHA against IgG4 F(ab’)_2_ generated by pepsin as an alternate protease of MMP-7, but not MMP-3, could be used as a potential diagnostic marker for RA, including seronegative RA.

## Supplementary information

**Additional file 1: Figure S1.** Time-course of human IgG1 (TCZ, IFX), IgG2 (PAN) and IgG4 (NTZ) digested by MMP-3. A main band of 150 kDa corresponding to intact IgG1 was depicted (lane 1: TCZ, lane 2: IFX). After 2 hours 125 kDa band of single cleaved IgG (scIgG), 100 kDa band of F(ab’)_2_ and 25 kDa band of Fc monomer (Fcm) were observed (lane 3: TCZ, lane 4: IFX, lane 6: NTZ) except IgG2. IgG2 showed only 125 kDa band as a digested fragment (lane 5). After 24 hours, 150 kDa band disappeared except IgG2 (lane 9), and increased staining intensities of both 100 and 25 kDa band were observed (lane 7: TCZ, lane 8: IFX, lane 10: NTZ). **Figure S2.** Human IgG1 fragments generated by proteolytic cleavage of MMP-3. Arrows represent the cleavage site between Pro232 and Glu233 (EU numbering) in the lower hinge domain. (A) 150 kDa intact IgG1, (B) 125 kDa single cleaved IgG (scIgG) fragment resulted from a single-proteolytic cleavage in one of the heavy chains in the lower hinge, followed by losing one Fc monomer (Fcm), (C) 100 kDa F(ab’)_2_ fragment caused by losing another Fcm, (D) 25 kDa Fcm. **Figure S3.** Comparison of AHA levels between non-RA and RA patients. AHA1 and AHA3 represent IgG AHA against TCZ IgG1 F(ab’)_2*pepsin*_ and NTZ IgG4 F(ab’)_2*pepsin*_, respectively. AHA5 and AHA7 represent IgA AHAs against TCZ IgG1 F(ab’)_2*pepsin*_ and NTZ IgG4 F(ab’)_2*pepsin*_, respectively. Differences in levels between non-RA and RA were analyzed with the Mann-Whitney U test.

## Data Availability

All data generated or analyzed during this study are included in this article and its supplementary information files.

## Abbreviations

ACPA: Anti-citrullinated protein antibodies
AHA: Anti-hinge antibody
ALP: Alkaline phosphatase
AU: Arbitrary units
DNRA: RF and anti-CCP2 double negative RA
DOR: Diagnostic odds ratio
DPRA: RF and anti-CCP2 double positive RA
EDTA: Ethylenediaminetetraacetic acid
ELISA: Enzyme-linked immunosorbent assay
HC: Healthy controls
IdeS: IgG-degrading enzyme of *Streptococcus pyogens*
IFX: Infliximab
LR+: Positive likelihood ratio
MMP-3: Matrix metalloproteinase-3
MMP-7: Matrix metalloproteinase-7
NTZ: Natalizumab
PAN: Panitumumab
PPV−: Negative predictive value
PPV+: Positive predictive value
RA: Rheumatoid arthritis
RF: Rheumatoid factor
ROC: Receiver operating characteristic
RT: Room temperature
SCB: Sodium citrate buffer
SDS-PAGE: Sodium dodecyl sulfate polyacrylamide gel electrophoresis
SPRA (CCP): RA with single positive anti-CCP2
SPRA (RF): RA with single positive RF
TBST: Tris-buffered saline with 0.05% Tween 20
TCZ: Tocilizumab
