# Supplementary figures and images for: IgG anti-hinge antibodies against IgG4 F(ab’)2 fragments generated using pepsin are useful diagnostic markers for rheumatoid arthritis: implications of the possible roles of metalloproteinases and IgG subclasses in generating immunogenic hinge epitopes
Source: Arthritis Res Ther. 2020 Jun 26;22:161. doi: 10.1186/s13075-020-02251-7 (PMC7318515; doi:10.1186/s13075-020-02251-7)

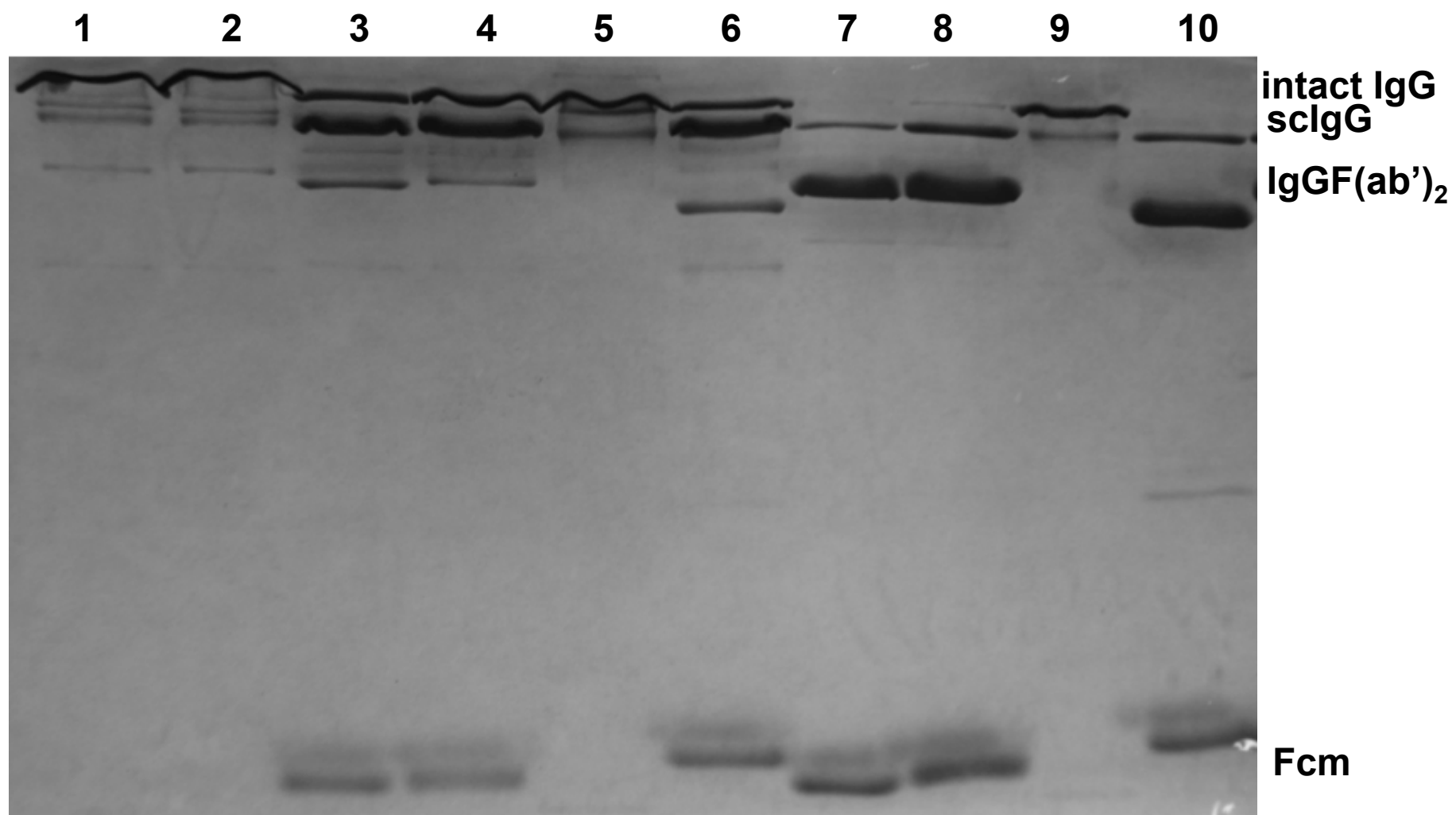

Supplement: Supplementary file 1 — Additional file 1: Figure S1. Time-course of human IgG1 (TCZ, IFX), IgG2 (PAN) and IgG4 (NTZ) digested by MMP-3. A main band of 150 kDa corresponding to intact IgG1 was depicted (lane 1: TCZ, lane 2: IFX). After 2 hours 125 kDa band of single cleaved IgG (scIgG), 100 kDa band of F(ab’)2 and 25 kDa band of Fc monomer (Fcm) were observed (lane 3: TCZ, lane 4: IFX, lane 6: NTZ) except IgG2. IgG2 showed only 125 kDa band as a digested fragment (lane 5). After 24 hours, 150 kDa band disappeared except IgG2 (lane 9), and increased staining intensities of both 100 and 25 kDa band were observed (lane 7: TCZ, lane 8: IFX, lane 10: NTZ). Figure S2. Human IgG1 fragments generated by proteolytic cleavage of MMP-3. Arrows represent the cleavage site between Pro232 and Glu233 (EU numbering) in the lower hinge domain. (A) 150 kDa intact IgG1, (B) 125 kDa single cleaved IgG (scIgG) fragment resulted from a single-proteolytic cleavage in one of the heavy chains in the lower hinge, followed by losing one Fc monomer (Fcm), (C) 100 kDa F(ab’)2 fragment caused by losing another Fcm, (D) 25 kDa Fcm. Figure S3. Comparison of AHA levels between non-RA and RA patients. AHA1 and AHA3 represent IgG AHA against TCZ IgG1 F(ab’)2pepsin and NTZ IgG4 F(ab’)2pepsin, respectively. AHA5 and AHA7 represent IgA AHAs against TCZ IgG1 F(ab’)2pepsin and NTZ IgG4 F(ab’)2pepsin, respectively. Differences in levels between non-RA and RA were analyzed with the Mann-Whitney U test. [file 13075_2020_2251_MOESM1_ESM.zip › Figure S1.pdf]

A

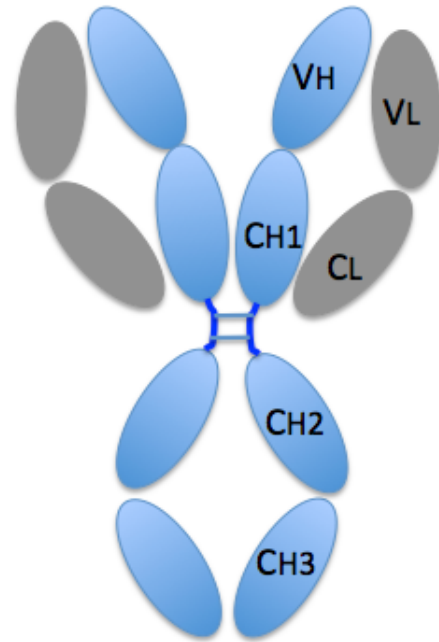

B

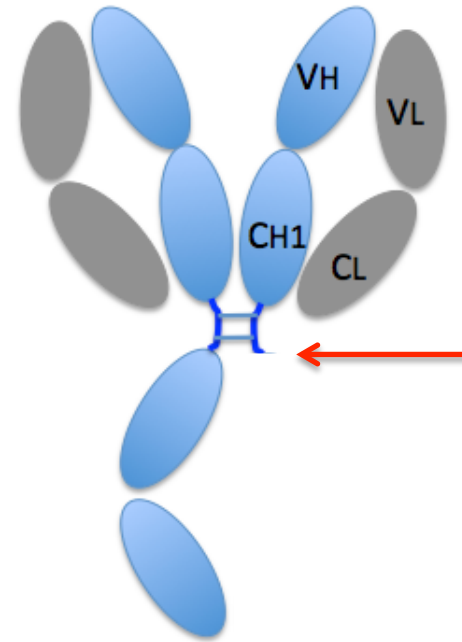

C

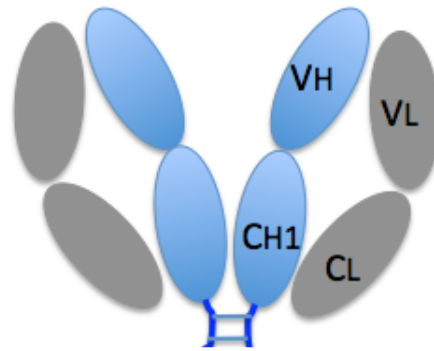

D

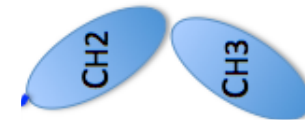

Supplement: Supplementary file 1 — Additional file 1: Figure S1. Time-course of human IgG1 (TCZ, IFX), IgG2 (PAN) and IgG4 (NTZ) digested by MMP-3. A main band of 150 kDa corresponding to intact IgG1 was depicted (lane 1: TCZ, lane 2: IFX). After 2 hours 125 kDa band of single cleaved IgG (scIgG), 100 kDa band of F(ab’)2 and 25 kDa band of Fc monomer (Fcm) were observed (lane 3: TCZ, lane 4: IFX, lane 6: NTZ) except IgG2. IgG2 showed only 125 kDa band as a digested fragment (lane 5). After 24 hours, 150 kDa band disappeared except IgG2 (lane 9), and increased staining intensities of both 100 and 25 kDa band were observed (lane 7: TCZ, lane 8: IFX, lane 10: NTZ). Figure S2. Human IgG1 fragments generated by proteolytic cleavage of MMP-3. Arrows represent the cleavage site between Pro232 and Glu233 (EU numbering) in the lower hinge domain. (A) 150 kDa intact IgG1, (B) 125 kDa single cleaved IgG (scIgG) fragment resulted from a single-proteolytic cleavage in one of the heavy chains in the lower hinge, followed by losing one Fc monomer (Fcm), (C) 100 kDa F(ab’)2 fragment caused by losing another Fcm, (D) 25 kDa Fcm. Figure S3. Comparison of AHA levels between non-RA and RA patients. AHA1 and AHA3 represent IgG AHA against TCZ IgG1 F(ab’)2pepsin and NTZ IgG4 F(ab’)2pepsin, respectively. AHA5 and AHA7 represent IgA AHAs against TCZ IgG1 F(ab’)2pepsin and NTZ IgG4 F(ab’)2pepsin, respectively. Differences in levels between non-RA and RA were analyzed with the Mann-Whitney U test. [file 13075_2020_2251_MOESM1_ESM.zip › Figure S2.pdf]

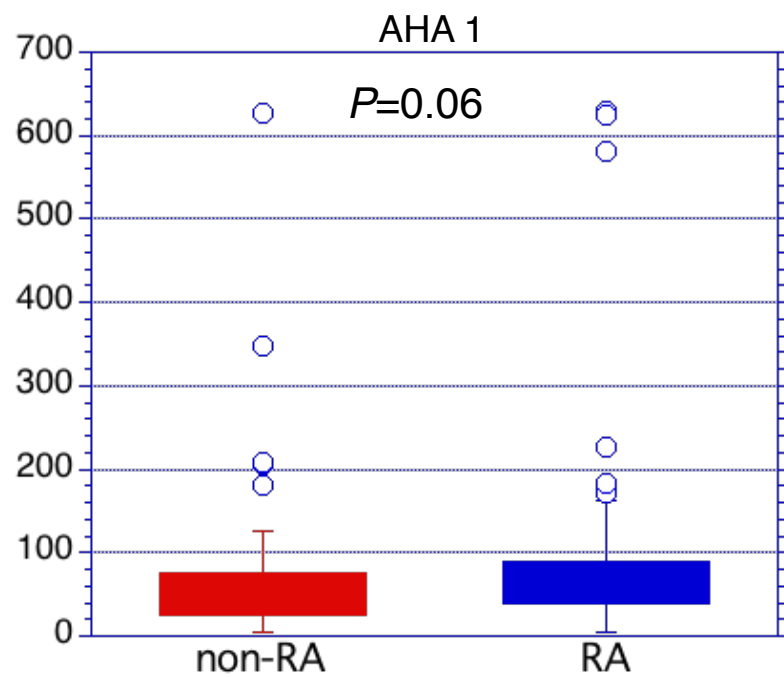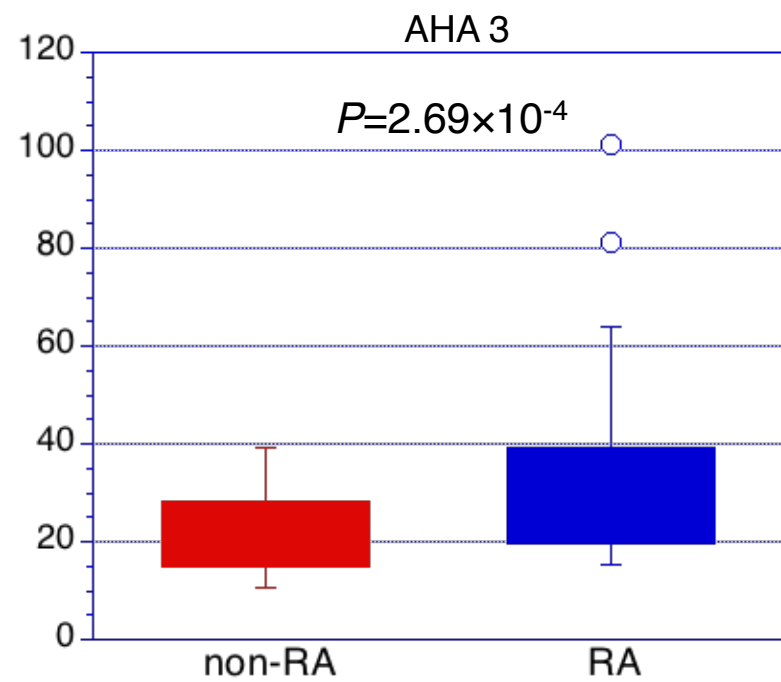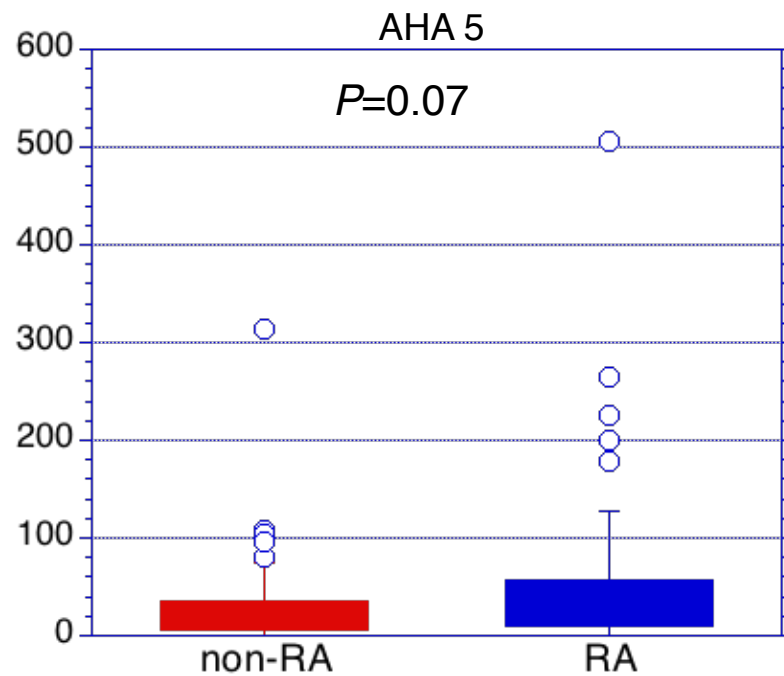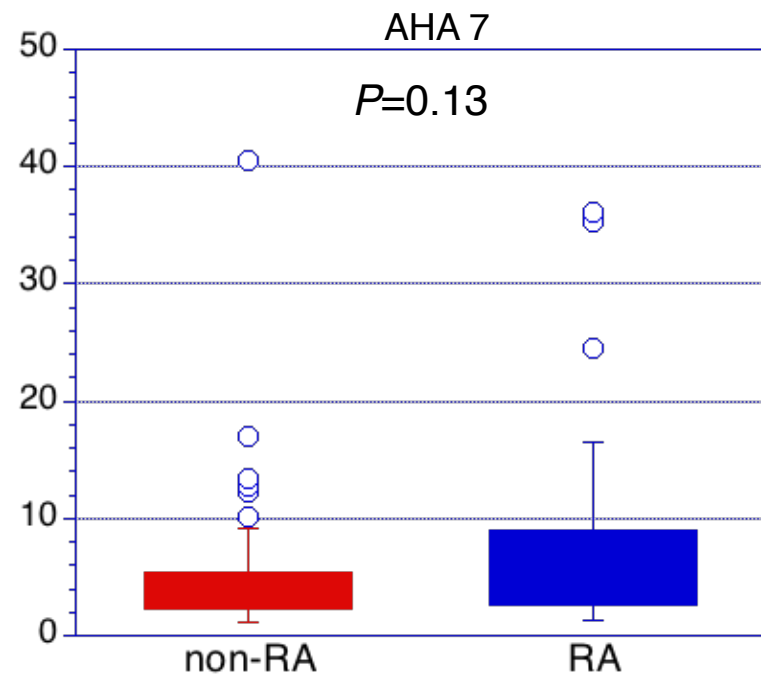

Supplement: Supplementary file 1 — Additional file 1: Figure S1. Time-course of human IgG1 (TCZ, IFX), IgG2 (PAN) and IgG4 (NTZ) digested by MMP-3. A main band of 150 kDa corresponding to intact IgG1 was depicted (lane 1: TCZ, lane 2: IFX). After 2 hours 125 kDa band of single cleaved IgG (scIgG), 100 kDa band of F(ab’)2 and 25 kDa band of Fc monomer (Fcm) were observed (lane 3: TCZ, lane 4: IFX, lane 6: NTZ) except IgG2. IgG2 showed only 125 kDa band as a digested fragment (lane 5). After 24 hours, 150 kDa band disappeared except IgG2 (lane 9), and increased staining intensities of both 100 and 25 kDa band were observed (lane 7: TCZ, lane 8: IFX, lane 10: NTZ). Figure S2. Human IgG1 fragments generated by proteolytic cleavage of MMP-3. Arrows represent the cleavage site between Pro232 and Glu233 (EU numbering) in the lower hinge domain. (A) 150 kDa intact IgG1, (B) 125 kDa single cleaved IgG (scIgG) fragment resulted from a single-proteolytic cleavage in one of the heavy chains in the lower hinge, followed by losing one Fc monomer (Fcm), (C) 100 kDa F(ab’)2 fragment caused by losing another Fcm, (D) 25 kDa Fcm. Figure S3. Comparison of AHA levels between non-RA and RA patients. AHA1 and AHA3 represent IgG AHA against TCZ IgG1 F(ab’)2pepsin and NTZ IgG4 F(ab’)2pepsin, respectively. AHA5 and AHA7 represent IgA AHAs against TCZ IgG1 F(ab’)2pepsin and NTZ IgG4 F(ab’)2pepsin, respectively. Differences in levels between non-RA and RA were analyzed with the Mann-Whitney U test. [file 13075_2020_2251_MOESM1_ESM.zip › Figure S3 .pdf]
